# 3-[(*E*)-(7-Chloro-4-quinol­yl)hydrazono­meth­yl]benzonitrile monohydrate

**DOI:** 10.1107/S1600536809050120

**Published:** 2009-11-28

**Authors:** Marcelle L. de Ferreira, Marcus V. N. de Souza, R. Alan Howie, Edward R. T. Tiekink, James L. Wardell, Solange M. S. V. Wardell

**Affiliations:** a Instituto de Tecnologia em Farmacos, Fundação Oswaldo Cruz (FIOCRUZ), Far-Manguinhos, Rua Sizenando Nabuco, 100 Manguinhos, 21041-250 Rio de Janeiro, RJ, Brazil; b Department of Chemistry, University of Aberdeen, Old Aberdeen, AB15 5NY, Scotland; c Department of Chemistry, University of Malaya, 50603 Kuala Lumpur, Malaysia; d Centro de Desenvolvimento Tecnológico em Saúde (CDTS), Fundação Oswaldo Cruz (FIOCRUZ), Casa Amarela, Campus de Manguinhos, Av. Brasil 4365, 21040-900 Rio de Janeiro, RJ, Brazil; e CHEMSOL, 1 Harcourt Road, Aberdeen AB15 5NY, Scotland

## Abstract

The title monohydrate, C_17_H_11_ClN_4_·H_2_O, features an essentially planar organic mol­ecule, as seen in the dihedral angle of 2.42 (8)° formed between the quinoline and benzene planes. The conformation about the imine bond is *E*, and the N—H group is oriented towards the quinoline residue. The major feature of the crystal packing is the formation of supra­molecular chains along [100], whereby the water mol­ecule accepts one N—H⋯O hydrogen bond and makes two O—H⋯N hydrogen bonds. A C—H⋯O link is also present.

## Related literature

For background information on the pharmacological activity of quinoline derivatives, see: Elslager *et al.* (1969 ▶); Font *et al.* (1997 ▶); Kaminsky & Meltzer (1968 ▶); Musiol *et al.* (2006 ▶); Nakamura *et al.* (1999 ▶); Palmer *et al.* (1993 ▶); Ridley (2002 ▶); Sloboda *et al.* (1991 ▶); Tanenbaum & Tuffanelli (1980 ▶); Warshakoon *et al.* (2006 ▶). For recent studies into quinoline-based anti-malarials, see: Andrade *et al.* (2007 ▶); Cunico *et al.* (2006 ▶); da Silva *et al.* (2003 ▶); de Souza *et al.* (2005 ▶). For a related crystallographic study on neutral species related to the title compound, see: Kaiser *et al.* (2009 ▶).
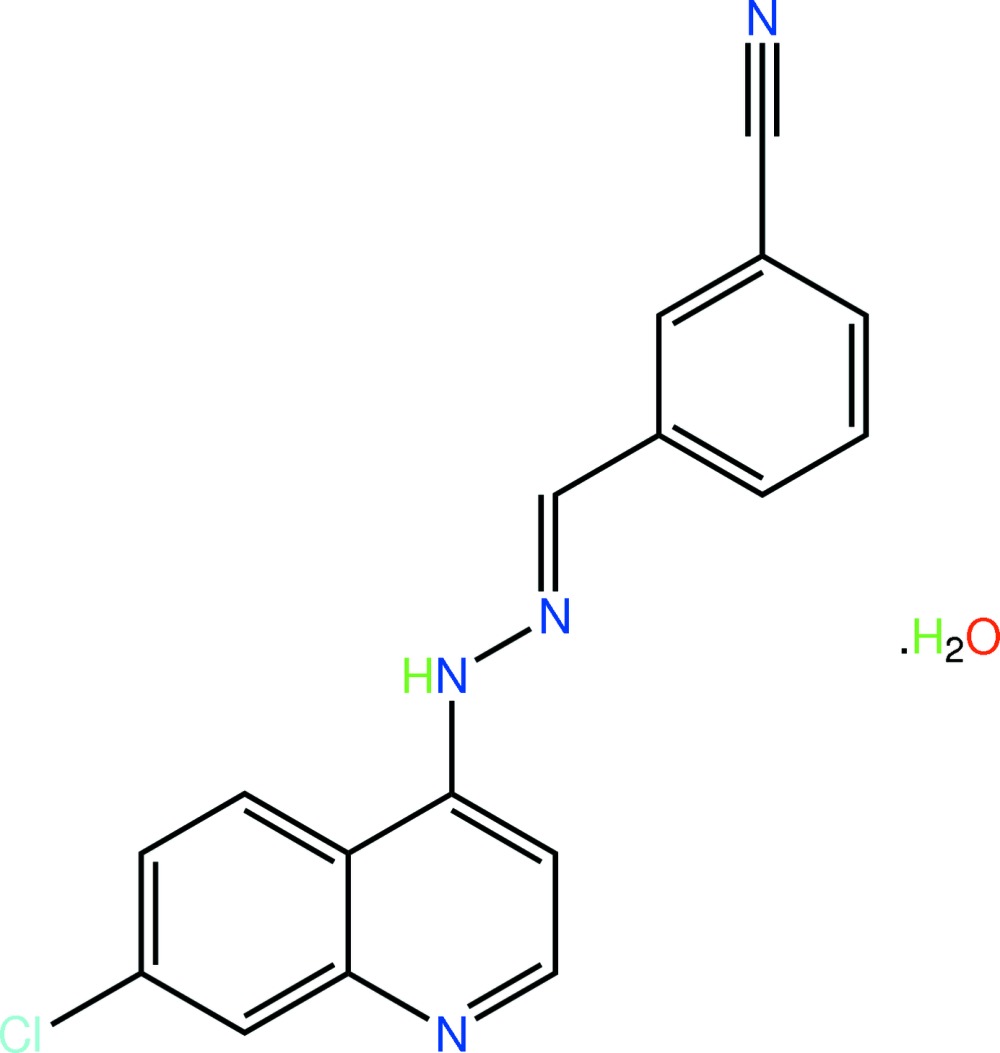


## Experimental

### 

#### Crystal data

C_17_H_11_ClN_4_·H_2_O
*M* *_r_* = 324.76
Triclinic, 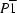

*a* = 8.7406 (2) Å
*b* = 9.8587 (3) Å
*c* = 10.2301 (2) Å
α = 110.8897 (15)°
β = 93.4341 (16)°
γ = 110.6766 (15)°
*V* = 752.93 (3) Å^3^
*Z* = 2
Mo *K*α radiation
μ = 0.26 mm^−1^
*T* = 120 K
0.12 × 0.09 × 0.04 mm

#### Data collection

Nonius KappaCCD area-detector diffractometer
Absorption correction: multi-scan (*SADABS*; Sheldrick, 2007 ▶) *T*_min_ = 0.901, *T*_max_ = 0.990
14600 measured reflections
3425 independent reflections
2902 reflections with *I* > 2σ(*I*)
*R*_int_ = 0.046

#### Refinement

*R*[*F* ^2^ > 2σ(*F* ^2^)] = 0.047
*wR*(*F* ^2^) = 0.109
*S* = 1.10
3425 reflections
217 parameters
H atoms treated by a mixture of independent and constrained refinement
Δρ_max_ = 0.32 e Å^−3^
Δρ_min_ = −0.25 e Å^−3^

### 

Data collection: *COLLECT* (Hooft, 1998 ▶); cell refinement: *DENZO* (Otwinowski & Minor, 1997 ▶) and *COLLECT*; data reduction: *DENZO* and *COLLECT*; program(s) used to solve structure: *SHELXS97* (Sheldrick, 2008 ▶); program(s) used to refine structure: *SHELXL97* (Sheldrick, 2008 ▶); molecular graphics: *DIAMOND* (Brandenburg, 2006 ▶); software used to prepare material for publication: *publCIF* (Westrip, 2009 ▶).

## Supplementary Material

Crystal structure: contains datablocks global, I. DOI: 10.1107/S1600536809050120/hb5242sup1.cif

Structure factors: contains datablocks I. DOI: 10.1107/S1600536809050120/hb5242Isup2.hkl

Additional supplementary materials:  crystallographic information; 3D view; checkCIF report

## Figures and Tables

**Table 1:** Hydrogen-bond geometry (Å, °)

*D*—H⋯*A*	*D*—H	H⋯*A*	*D*⋯*A*	*D*—H⋯*A*
O1w—H1w⋯N4^i^	0.83 (3)	2.21 (3)	2.982 (3)	155 (3)
O1w—H2w⋯N1^ii^	0.86 (3)	1.99 (3)	2.828 (3)	164 (3)
N2—H2n⋯O1w	0.86 (3)	2.07 (3)	2.917 (2)	167 (3)
C5—H5⋯O1w	0.95	2.39	3.331 (3)	169

Symmetry codes: (i) 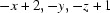; (ii) 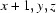.

**Fig. 1.**
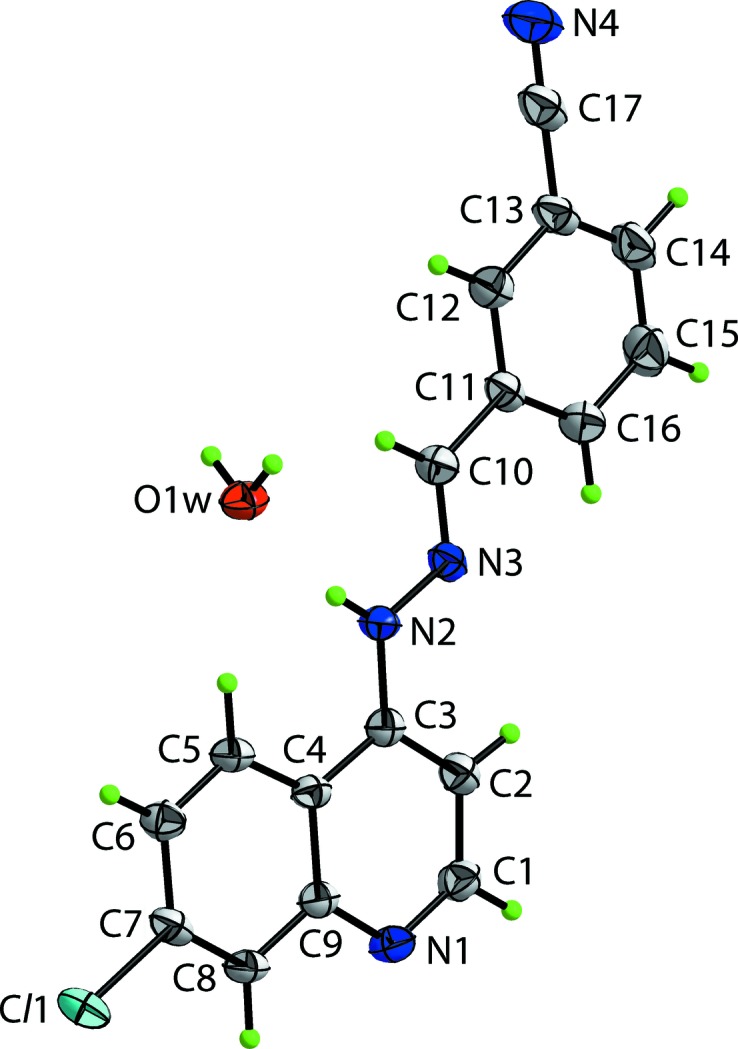
The asymmetric unit in (I) showing displacement ellipsoids at the 70% probability level.

**Fig. 2.**
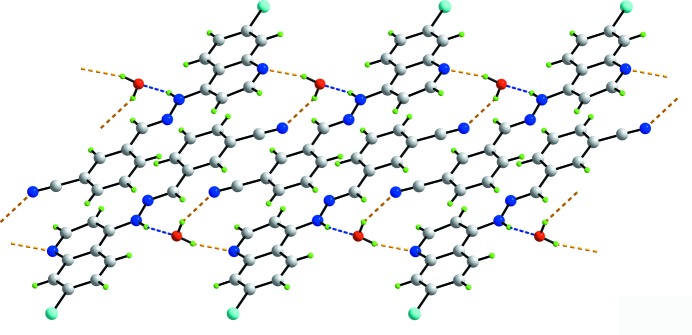
Supramolecular 1-D chain in (I) aligned along [100]. The O–H···N (orange) and N–H···O (blue) hydrogen bonds are shown as dashed lines. Colour code: Cl, cyan; O, red; N, blue; C, grey; and H, green.

## supplementary crystallographic information

### Comment

The title compound, crystallized as a hydrate, (I), was prepared as part of an on-going investigation aimed at developing anti-malarial compounds based on the quinoline nucleus (Andrade *et al.*,2007; Cunico *et al.*, 2006; da Silva *et al.*, 2003; de Souza *et al.*, 2005. The motivation for examining quinoline derivatives arises as the majority of anti-malarial drugs, such as chloroquine (Tanenbaum & Tuffanelli, 1980), mefloquine (Palmer *et al.*, 1993), primaquine (Elslager *et al.*, 1969) and amodiaquine (Ridley, 2002), possess a quinoline ring which has been the mainstay of malaria chemotherapy for much of the past 40 years (Font *et al.*, 1997; Kaminsky & Meltzer, 1968; Musiol *et al.*, 2006; Nakamura *et al.*, 1999; Sloboda *et al.*, 1991; Warshakoon *et al.*, 2006).

The molecular structure of (I), Fig. 1, comprises an essentially planar quinoline framework with the maximum deviation from the least-squares plane through the non-hydrogen atoms being -0.012 (2) Å for atom C3. The planarity extends through the azo moiety (the C2–C3–N2–N3 and N2–N3–C10–C11 torsion angles are 2.4 (3) and -179.76 (16) °, respectively) into the terminal benzene; the dihedral angle formed between the quinoline and benzene rings is 2.42 (8) °. The conformation about the C10N3 bond is *E*. Finally, the amine-N2 group is oriented towards the quinoline nucleus as observed in related structures (Kaiser *et al.*, (2009).

The water molecule of crystallization plays a pivotal role in the crystal packing. The water-H atoms form hydrogen bonds to the the pyridine-N1 and nitrile-N4 atoms, derived from different molecules, and at the same time accepts a hydrogen bond from the amino-N2 atom, Table 1. The tetrahedral environment for the O1w atom is completed by an acceptor interaction from the C5—H atom. The net result of the hydrogen bonding interactions is the formation of a supramolecular chain aligned along the *a* direction, Fig. 2.

### Experimental

A solution of 7-chloro-4-hydrazinoquinoline (0.20 g, 1.0 mmol) and 3-cyanobenzaldehyde (1.2 mmol) in EtOH (5 ml) was maintained at room temperature overnight and rotary evaporated. The solid residue, was washed with cold Et_2_O (3 *x* 10 ml) and recrystallized from EtOH m. pt. 485–487 K, yield 77%. The sample for the X-ray study was slowly grown from moist EtOH and was found to be the monohydrate. MS/ESI: 305 [M—H—H_2_O], based on ^35^Cl. IR [KBr, cm^-1^] ν: 3215 (NH), 1577 (CN).

### Refinement

The C-bound H atoms were geometrically placed (C–H = 0.95 Å) and refined as riding with *U*_iso_(H) = 1.2*U*_eq_(C). The O– and N-bound H atoms were located from a difference map and refined with *U*_iso_(H) = 1.2*U*_eq_(N) or 1.5*U*_eq_(O).

### Crystal data

**Table:**

C_17_H_11_ClN_4_·H_2_O	*Z* = 2
*M**_r_* = 324.76	*F*(000) = 336
Triclinic, *P*1	*D*_x_ = 1.432 Mg m^−^^3^
Hall symbol: -P 1	Mo *K*α radiation, λ = 0.71073 Å
*a* = 8.7406 (2) Å	Cell parameters from 11816 reflections
*b* = 9.8587 (3) Å	θ = 2.9–27.5°
*c* = 10.2301 (2) Å	µ = 0.26 mm^−^^1^
α = 110.8897 (15)°	*T* = 120 K
β = 93.4341 (16)°	Plate, yellow
γ = 110.6766 (15)°	0.12 × 0.09 × 0.04 mm
*V* = 752.93 (3) Å^3^	

### Data collection

**Table:**

Nonius KappaCCD area-detector diffractometer	3425 independent reflections
Radiation source: Enraf Nonius FR591 rotating anode	2902 reflections with *I* > 2σ(*I*)
10 cm confocal mirrors	*R*_int_ = 0.046
Detector resolution: 9.091 pixels mm^-1^	θ_max_ = 27.5°, θ_min_ = 3.0°
φ and ω scans	*h* = −11→11
Absorption correction: multi-scan (*SADABS*; Sheldrick, 2007)	*k* = −12→12
*T*_min_ = 0.901, *T*_max_ = 0.990	*l* = −13→13
14600 measured reflections	

### Refinement

**Table:**

Refinement on *F*^2^	Primary atom site location: structure-invariant direct methods
Least-squares matrix: full	Secondary atom site location: difference Fourier map
*R*[*F*^2^ > 2σ(*F*^2^)] = 0.047	Hydrogen site location: inferred from neighbouring sites
*wR*(*F*^2^) = 0.109	H atoms treated by a mixture of independent and constrained refinement
*S* = 1.10	*w* = 1/[σ^2^(*F*_o_^2^) + (0.023*P*)^2^ + 0.7336*P*] where *P* = (*F*_o_^2^ + 2*F*_c_^2^)/3
3425 reflections	(Δ/σ)_max_ < 0.001
217 parameters	Δρ_max_ = 0.32 e Å^−^^3^
0 restraints	Δρ_min_ = −0.25 e Å^−^^3^

### Special details

**Table:**

Geometry. All s.u.'s (except the s.u. in the dihedral angle between two l.s. planes) are estimated using the full covariance matrix. The cell s.u.'s are taken into account individually in the estimation of s.u.'s in distances, angles and torsion angles; correlations between s.u.'s in cell parameters are only used when they are defined by crystal symmetry. An approximate (isotropic) treatment of cell s.u.'s is used for estimating s.u.'s involving l.s. planes.
Refinement. Refinement of *F*^2^ against ALL reflections. The weighted *R*-factor *wR* and goodness of fit *S* are based on *F*^2^, conventional *R*-factors *R* are based on *F*, with *F* set to zero for negative *F*^2^. The threshold expression of *F*^2^ > 2σ(*F*^2^) is used only for calculating *R*-factors(gt) *etc*. and is not relevant to the choice of reflections for refinement. *R*-factors based on *F*^2^ are statistically about twice as large as those based on *F*, and *R*- factors based on ALL data will be even larger.

### Fractional atomic coordinates and isotropic or equivalent isotropic displacement parameters (Å^2^)

**Table:**

	*x*	*y*	*z*	*U*_iso_*/*U*_eq_	
Cl1	0.19140 (6)	0.44486 (6)	−0.08216 (5)	0.02503 (14)	
N1	0.0883 (2)	0.39813 (19)	0.38535 (17)	0.0215 (3)	
N2	0.49296 (19)	0.27107 (18)	0.43632 (17)	0.0181 (3)	
H2N	0.559 (3)	0.270 (3)	0.377 (2)	0.022*	
N3	0.52218 (19)	0.23502 (18)	0.55031 (16)	0.0181 (3)	
N4	1.1329 (2)	0.0074 (2)	0.7938 (2)	0.0357 (5)	
C1	0.1296 (2)	0.3704 (2)	0.4975 (2)	0.0217 (4)	
H1	0.0635	0.3806	0.5679	0.026*	
C2	0.2613 (2)	0.3278 (2)	0.5209 (2)	0.0204 (4)	
H2	0.2816	0.3086	0.6036	0.024*	
C3	0.3625 (2)	0.3139 (2)	0.42154 (19)	0.0164 (4)	
C4	0.3262 (2)	0.3449 (2)	0.29879 (19)	0.0160 (4)	
C5	0.4207 (2)	0.3368 (2)	0.1905 (2)	0.0191 (4)	
H5	0.5150	0.3113	0.1983	0.023*	
C6	0.3779 (2)	0.3654 (2)	0.0746 (2)	0.0203 (4)	
H6	0.4411	0.3583	0.0019	0.024*	
C7	0.2398 (2)	0.4052 (2)	0.0646 (2)	0.0194 (4)	
C8	0.1449 (2)	0.4147 (2)	0.1657 (2)	0.0193 (4)	
H8	0.0515	0.4409	0.1557	0.023*	
C9	0.1869 (2)	0.3852 (2)	0.28647 (19)	0.0178 (4)	
C10	0.6454 (2)	0.1947 (2)	0.5564 (2)	0.0197 (4)	
H10	0.7095	0.1919	0.4844	0.024*	
C11	0.6892 (2)	0.1527 (2)	0.67264 (19)	0.0192 (4)	
C12	0.8252 (2)	0.1113 (2)	0.6757 (2)	0.0216 (4)	
H12	0.8867	0.1086	0.6020	0.026*	
C13	0.8715 (2)	0.0735 (2)	0.7873 (2)	0.0231 (4)	
C14	0.7821 (3)	0.0759 (2)	0.8950 (2)	0.0286 (5)	
H14	0.8144	0.0508	0.9708	0.034*	
C15	0.6451 (3)	0.1154 (3)	0.8908 (2)	0.0298 (5)	
H15	0.5829	0.1166	0.9640	0.036*	
C16	0.5979 (3)	0.1531 (2)	0.7807 (2)	0.0240 (4)	
H16	0.5034	0.1792	0.7786	0.029*	
C17	1.0162 (3)	0.0351 (2)	0.7902 (2)	0.0275 (5)	
O1W	0.74203 (19)	0.2487 (2)	0.26148 (16)	0.0261 (3)	
H1W	0.746 (3)	0.160 (3)	0.231 (3)	0.039*	
H2W	0.845 (4)	0.311 (3)	0.302 (3)	0.039*	

### Atomic displacement parameters (Å^2^)

**Table:**

	*U*^11^	*U*^22^	*U*^33^	*U*^12^	*U*^13^	*U*^23^
Cl1	0.0233 (2)	0.0305 (3)	0.0261 (3)	0.0115 (2)	0.00062 (18)	0.0168 (2)
N1	0.0203 (8)	0.0239 (8)	0.0255 (8)	0.0132 (7)	0.0078 (7)	0.0109 (7)
N2	0.0171 (8)	0.0231 (8)	0.0199 (8)	0.0113 (6)	0.0052 (6)	0.0115 (7)
N3	0.0198 (8)	0.0162 (7)	0.0177 (7)	0.0070 (6)	0.0004 (6)	0.0069 (6)
N4	0.0342 (10)	0.0308 (10)	0.0417 (11)	0.0172 (9)	−0.0047 (9)	0.0122 (9)
C1	0.0209 (9)	0.0243 (10)	0.0229 (9)	0.0116 (8)	0.0080 (8)	0.0097 (8)
C2	0.0210 (9)	0.0211 (9)	0.0201 (9)	0.0087 (8)	0.0031 (7)	0.0094 (8)
C3	0.0150 (8)	0.0130 (8)	0.0191 (9)	0.0044 (7)	0.0018 (7)	0.0055 (7)
C4	0.0144 (8)	0.0136 (8)	0.0195 (9)	0.0063 (7)	0.0021 (7)	0.0056 (7)
C5	0.0162 (9)	0.0211 (9)	0.0225 (9)	0.0100 (7)	0.0031 (7)	0.0094 (8)
C6	0.0182 (9)	0.0218 (9)	0.0223 (9)	0.0083 (8)	0.0049 (7)	0.0100 (8)
C7	0.0188 (9)	0.0179 (9)	0.0217 (9)	0.0067 (7)	−0.0013 (7)	0.0097 (7)
C8	0.0147 (8)	0.0189 (9)	0.0250 (9)	0.0084 (7)	0.0012 (7)	0.0085 (8)
C9	0.0153 (8)	0.0155 (8)	0.0212 (9)	0.0068 (7)	0.0020 (7)	0.0055 (7)
C10	0.0192 (9)	0.0211 (9)	0.0206 (9)	0.0089 (7)	0.0036 (7)	0.0095 (8)
C11	0.0207 (9)	0.0156 (8)	0.0191 (9)	0.0061 (7)	−0.0013 (7)	0.0066 (7)
C12	0.0212 (9)	0.0182 (9)	0.0236 (9)	0.0066 (8)	0.0017 (8)	0.0085 (8)
C13	0.0247 (10)	0.0163 (9)	0.0250 (10)	0.0077 (8)	−0.0045 (8)	0.0069 (8)
C14	0.0397 (12)	0.0246 (10)	0.0222 (10)	0.0140 (9)	−0.0016 (9)	0.0107 (8)
C15	0.0408 (12)	0.0311 (11)	0.0229 (10)	0.0172 (10)	0.0082 (9)	0.0138 (9)
C16	0.0272 (10)	0.0246 (10)	0.0230 (10)	0.0134 (8)	0.0045 (8)	0.0101 (8)
C17	0.0321 (11)	0.0200 (10)	0.0266 (10)	0.0093 (9)	−0.0053 (9)	0.0082 (8)
O1W	0.0213 (7)	0.0359 (8)	0.0266 (8)	0.0169 (7)	0.0065 (6)	0.0130 (7)

### Geometric parameters (Å, °)

**Table:**

Cl1—C7	1.7454 (18)	C7—C8	1.362 (3)
N1—C1	1.328 (2)	C8—C9	1.423 (2)
N1—C9	1.368 (2)	C8—H8	0.9500
N2—C3	1.366 (2)	C10—C11	1.462 (2)
N2—N3	1.369 (2)	C10—H10	0.9500
N2—H2N	0.86 (2)	C11—C12	1.388 (3)
N3—C10	1.278 (2)	C11—C16	1.401 (3)
N4—C17	1.147 (3)	C12—C13	1.401 (3)
C1—C2	1.393 (3)	C12—H12	0.9500
C1—H1	0.9500	C13—C14	1.386 (3)
C2—C3	1.389 (3)	C13—C17	1.444 (3)
C2—H2	0.9500	C14—C15	1.387 (3)
C3—C4	1.437 (2)	C14—H14	0.9500
C4—C5	1.417 (3)	C15—C16	1.389 (3)
C4—C9	1.419 (2)	C15—H15	0.9500
C5—C6	1.373 (3)	C16—H16	0.9500
C5—H5	0.9500	O1W—H1W	0.83 (3)
C6—C7	1.403 (3)	O1W—H2W	0.86 (3)
C6—H6	0.9500		
			
C1—N1—C9	116.07 (16)	C9—C8—H8	120.3
C3—N2—N3	119.16 (15)	N1—C9—C4	123.72 (16)
C3—N2—H2N	121.4 (14)	N1—C9—C8	116.93 (16)
N3—N2—H2N	119.4 (14)	C4—C9—C8	119.35 (16)
C10—N3—N2	115.64 (16)	N3—C10—C11	120.96 (17)
N1—C1—C2	125.78 (18)	N3—C10—H10	119.5
N1—C1—H1	117.1	C11—C10—H10	119.5
C2—C1—H1	117.1	C12—C11—C16	119.17 (17)
C3—C2—C1	118.91 (17)	C12—C11—C10	118.91 (17)
C3—C2—H2	120.5	C16—C11—C10	121.92 (17)
C1—C2—H2	120.5	C11—C12—C13	120.01 (18)
N2—C3—C2	122.36 (16)	C11—C12—H12	120.0
N2—C3—C4	119.55 (16)	C13—C12—H12	120.0
C2—C3—C4	118.09 (16)	C14—C13—C12	120.63 (18)
C5—C4—C9	118.81 (16)	C14—C13—C17	120.38 (17)
C5—C4—C3	123.77 (16)	C12—C13—C17	118.98 (19)
C9—C4—C3	117.42 (16)	C13—C14—C15	119.29 (18)
C6—C5—C4	120.96 (17)	C13—C14—H14	120.4
C6—C5—H5	119.5	C15—C14—H14	120.4
C4—C5—H5	119.5	C14—C15—C16	120.6 (2)
C5—C6—C7	119.30 (18)	C14—C15—H15	119.7
C5—C6—H6	120.4	C16—C15—H15	119.7
C7—C6—H6	120.4	C15—C16—C11	120.31 (19)
C8—C7—C6	122.12 (17)	C15—C16—H16	119.8
C8—C7—Cl1	119.72 (14)	C11—C16—H16	119.8
C6—C7—Cl1	118.16 (15)	N4—C17—C13	178.8 (2)
C7—C8—C9	119.46 (16)	H1W—O1W—H2W	102 (2)
C7—C8—H8	120.3		
			
C3—N2—N3—C10	179.41 (16)	C5—C4—C9—N1	−179.23 (17)
C9—N1—C1—C2	−1.0 (3)	C3—C4—C9—N1	1.1 (3)
N1—C1—C2—C3	0.9 (3)	C5—C4—C9—C8	0.6 (3)
N3—N2—C3—C2	2.4 (3)	C3—C4—C9—C8	−179.14 (16)
N3—N2—C3—C4	−177.19 (15)	C7—C8—C9—N1	179.30 (17)
C1—C2—C3—N2	−179.34 (17)	C7—C8—C9—C4	−0.5 (3)
C1—C2—C3—C4	0.2 (3)	N2—N3—C10—C11	−179.76 (16)
N2—C3—C4—C5	−1.2 (3)	N3—C10—C11—C12	−179.33 (17)
C2—C3—C4—C5	179.21 (17)	N3—C10—C11—C16	0.7 (3)
N2—C3—C4—C9	178.48 (16)	C16—C11—C12—C13	−1.2 (3)
C2—C3—C4—C9	−1.1 (2)	C10—C11—C12—C13	178.81 (17)
C9—C4—C5—C6	−0.7 (3)	C11—C12—C13—C14	0.4 (3)
C3—C4—C5—C6	178.94 (17)	C11—C12—C13—C17	−178.30 (17)
C4—C5—C6—C7	0.8 (3)	C12—C13—C14—C15	0.4 (3)
C5—C6—C7—C8	−0.8 (3)	C17—C13—C14—C15	179.10 (19)
C5—C6—C7—Cl1	178.69 (14)	C13—C14—C15—C16	−0.4 (3)
C6—C7—C8—C9	0.6 (3)	C14—C15—C16—C11	−0.4 (3)
Cl1—C7—C8—C9	−178.84 (14)	C12—C11—C16—C15	1.2 (3)
C1—N1—C9—C4	0.0 (3)	C10—C11—C16—C15	−178.82 (19)
C1—N1—C9—C8	−179.86 (17)		

### Hydrogen-bond geometry (Å, °)

**Table:**

*D*—H···*A*	*D*—H	H···*A*	*D*···*A*	*D*—H···*A*
O1w—H1w···N4^i^	0.83 (3)	2.21 (3)	2.982 (3)	155 (3)
O1w—H2w···N1^ii^	0.86 (3)	1.99 (3)	2.828 (3)	164 (3)
N2—H2n···O1w	0.86 (3)	2.07 (3)	2.917 (2)	167 (3)
C5—H5···O1w	0.95	2.39	3.331 (3)	169

Symmetry codes: (i) −*x*+2, −*y*, −*z*+1; (ii) *x*+1, *y*, *z*.
